# Time Variability of C-Reactive Protein: Implications for Clinical Risk Stratification

**DOI:** 10.1371/journal.pone.0060759

**Published:** 2013-04-08

**Authors:** Peter Bogaty, Gilles R. Dagenais, Lawrence Joseph, Luce Boyer, Anne Leblanc, Patrick Bélisle, James M. Brophy

**Affiliations:** 1 Quebec Heart and Lung Institute, Laval University, Quebec City, Quebec, Canada; 2 McGill University Health Center, Montreal, Quebec, Canada; University of Leicester, United Kingdom

## Abstract

**Background:**

C-reactive protein (CRP) is proposed as a screening test for predicting risk and guiding preventive approaches in coronary artery disease (CAD). However, the stability of repeated CRP measurements over time in subjects with and without CAD is not well defined. We sought to determine the stability of serial CRP measurements in stable subjects with distinct CAD manifestations and a group without CAD while carefully controlling for known confounders.

**Methods:**

We prospectively studied 4 groups of 25 stable subjects each 1) a history of recurrent acute coronary events; 2) a single myocardial infarction ≥7 years ago; 3) longstanding CAD (≥7 years) that had never been unstable; 4) no CAD. Fifteen measurements of CRP were obtained to cover 21 time-points: 3 times during one day; 5 consecutive days; 4 consecutive weeks; 4 consecutive months; and every 3 months over the year. CRP risk threshold was set at 2.0 mg/L. We estimated variance across time-points using standard descriptive statistics and Bayesian hierarchical models.

**Results:**

Median CRP values of the 4 groups and their pattern of variability did not differ substantially so all subjects were analyzed together. The median individual standard deviation (SD) CRP values within-day, within-week, between-weeks and between-months were 0.07, 0.19, 0.36 and 0.63 mg/L, respectively. Forty-six percent of subjects changed CRP risk category at least once and 21% had ≥4 weekly and monthly CRP values in both low and high-risk categories.

**Conclusions:**

Considering its large intra-individual variability, it may be problematic to rely on CRP values for CAD risk prediction and therapeutic decision-making in individual subjects.

## Introduction

The pathophysiological contribution of inflammation to atherosclerotic disease is well recognized and blood-borne C-reactive protein (CRP) is a well-known non-specific indicator of inflammatory status. [1]–[3] Elevated levels of CRP have been associated with increased long-term risk of developing clinical manifestations of atherosclerotic disease in primary [4], [5] and secondary prevention studies [6] although the incremental value of CRP for predicting risk, monitoring risk reduction and guiding treatment remains controversial. [7]–[11] Notwithstanding this uncertainty, there is increasing support for the clinical utility of CRP for risk prediction and for guiding preventive approaches [12], [13].

Previous studies that have addressed the stability of CRP measurements within individuals over time are conflicting, [14]–[23] have not evaluated the complete spectrum of patients and have not extensively examined reproducibility while controlling for potentially confounding variables. Therefore, we undertook this study to prospectively determine the stability of serial CRP measurements over one year in stable subjects with several distinct manifestations of coronary artery disease (CAD) and in a group without CAD while carefully controlling for known confounders. We based ourselves on previous work in which we found differences in biomarker patterns (albeit only measured once) in similar subsets of subjects [24].

## Methods

### Patients

We recruited 4 groups of 25 stable subjects each (a convenience sample) who had either: 1) a history of recurrent (≥3) acute coronary events (unstable angina or myocardial infarction [MI] with at least 2 of the latter) with the last event within 3 years but >3 months prior to blood sampling; 2) a single remote MI ≥7 years previously; 3) longstanding (≥7 years) stable CAD without previous acute instability; 4) no CAD; these latter subjects were sex and age-matched (within one year) with subjects in one of the other groups and had to have an unequivocally normal coronary angiogram performed within 3 years of blood sampling and no evidence of any vascular disease. The study subjects were identified in a tertiary cardiac hospital by scanning consecutive discharge summaries of patients hospitalized with a diagnosis of MI or unstable angina in the preceding 5 years and by scanning the notes of consecutive patients at the cardiac outpatient clinic or undergoing coronary angiography between 2005 and 2008.

At the time of first blood sampling, there had to be no ongoing or recent (<1 month) inflammatory/infectious disease, no surgical procedure or angioplasty in the preceding 3 months and no angiography in the preceding month.

This study complies with the Declaration of Helsinki. It was approved by the hospital ethics committee (‘Comité d’éthique de la recherché de l’Institut universitaire de cardiologie et de pneumologie de Québec’) and each participant gave written informed consent.

### Study Procedures

After recruitment, subjects had fasting baseline blood tests, including CRP. A schedule of subsequent blood measurement dates was adapted to each subject’s availability. At each visit, subjects underwent a detailed structured questionnaire and drug history whose object was to determine any events or factors that could impact on inflammatory status to minimize any systematic variability in CRP. Three blood samples for measuring CRP were collected during a single day at 6–8 hour intervals. In addition, there were: 1) 5 daily blood samples on consecutive days; 2) 4 consecutive weekly samples; 3) 4 consecutive monthly samples; and 4) tri-monthly samples to complete the year. Blood sampling at any single time could count more than once in these determinations of diurnal, daily, weekly, monthly and tri-monthly variability so that the 21 measuring time-points were covered by 15 blood samples. Blood samples were centrifuged, distributed in appropriate aliquots and frozen at −80°C. After the last blood sample was collected in the last subject, all CRP measurements were performed simultaneously. CRP was measured in heparinized plasma with the N High Sensitivity CRP assay (inter-assay reproducibility 4.7%, assay range 0.18–1100 mg/L, sensitivity 0.18 mg/L) using the Behring ProSpec Nephelometer Analyzer (Siemens Healthcare Diagnosis, Deerfield, IL).

If any study subject had a significant inflammatory event like fever or a hospitalization on the designated blood sampling day that could impact on CRP values, blood sampling was either delayed or cancelled, depending on whether the required measurement could or could not be performed with respect to appropriate adherence to the study protocol and the measuring intervals. For example, if this event occurred during the 5 daily measurements, a day could be lost (and become missing data) or the 5 sampling days could be moved to another time during the year. At the other extreme, a tri-monthly measurement that could be problematic because of a concomitant condition could be postponed up to 2 weeks later and still be counted. Patients were asked to report any active or recent symptom or event at each blood sampling time. Examples include musculoskeletal symptoms, ongoing or recent upper respiratory infection, significant headaches, recent vaccination, change in medication, death of someone close, depression, and marked change in exercise status or alcohol consumption. Before blood analyses were performed, the investigators adjudicated all such reported events as representing or not a potential confounder of inflammatory status qualified as mild, moderate, or severe.

In the qualitative analysis of CRP results, we considered values ≥2 mg/L as indicative of high risk and values <2 mg/L as low risk.

### Statistical Methods

Investigating the variability of CRP across time can be done using different statistical measures. Some authors have used correlation coefficients, but even very high values of the correlation do not necessarily imply low variability. [18], [21], [23] Similarly, authors have used intra-class correlation coefficients [19], [23] **,** but these are also not optimal, since they are defined as a ratio of between-group variance to total variance, and therefore not a direct measure of within-individual variability. We therefore chose to directly report the variance of CRP, both in terms of descriptive statistics for different time periods, and as estimated by a Bayesian hierarchical model, described below.

The design of the study allowed for estimating the variance of CRP across different time periods, including variability within one day, across several consecutive days, across weeks, and across months. Our analysis took advantage of this design, estimating CRP variability in 3 different ways.

First, we compiled descriptive statistics for all variables, including means and standard deviations (SD) of CRP, and percentages of baseline categorical variables. Included in these descriptive statistics were estimates of the SDs for each time interval of interest, calculated directly using the observations from the relevant time period. These were done both assuming a common SD across all individuals for each time period and allowing individual specific SDs at each time period. In the latter case, we calculated the SD for each individual, and report the median SD value across individuals. To compare CRP values across the 4 clinical groups, medians within each group were calculated by first taking the median value within each subject, and then taking the median across subjects in each group. Confidence intervals (CI) were calculated for the medians within each group.

Second, while it may be reasonable to assume that each individual has a constant global CRP mean over time, varying only randomly, it is also possible that homoeostatic imbalances cause this mean to shift slightly over days, weeks, or months. Variations could also most likely be due to some combination of these two effects. We therefore constructed a hierarchical model with five different time levels, wherein each individual was allowed to have his or her own mean that could also vary over each time interval. This model will provide conservative estimates of variability compared to a model that forces a fixed mean across time within each subject and which considers all variation to be purely random. This would imply that for each subject if an infinite number of readings were available at each time-point, the averages would be identical. This seems unrealistic and explains why we have chosen a hierarchical approach.

Specifically, for each individual, the first level of the hierarchical model assumed a normal density for their CRP values within each day. At the second level of the hierarchical model, the individual within-day means followed a normal density, with the mean of this density allowed to vary by week. Similarly, a third level was added to accommodate monthly variations. At the fourth level of our model, variations between monthly means across individuals followed a normal density, with a global mean per individual. At the top level of our hierarchical model, individual means were assumed to follow a normal density, with a global mean. While means can vary within individuals over time, our model ensures that any such changes will arise only from strong evidence in the data, otherwise the hierarchical structure will tend to pull means back to their overall averages. The variances estimated from these models were similarly ordered in a hierarchical fashion. In particular, the variance within days was nested into the variance within weeks, and then within months. Our global mean was given a very diffuse prior distribution, and similarly, all SDs from the above densities were given very wide uniform priors, covering the range of all plausible values with equal probability. Therefore, all inferences are essentially driven by the observed data. Models were fit for the study sample as a whole, and also within subgroups of subjects taking or not taking lipid-lowering medications.

Finally, we fit another hierarchical model similar to the above, but now adding in potential covariates to attempt to explain between subject variability. Potential covariates, selected initially for potential effects from a clinical viewpoint, included aspirin, body mass index (BMI), sex, clinical group, left ventricular ejection fraction, use of lipid-lowering drugs and angiotensin-converting-enzyme inhibitors and adjudicated inflammation status. Final variable selection was by the BIC criterion. [25] All results are provided with 95% confidence intervals (CI) for frequentist results, and 95% credible intervals (CrI) for all Bayesian models. Models were fit using WinBUGS (Version 1.4.3, Cambridge, UK). The details of our approach with mathematical notation that describes exactly what is in each of the 5 levels of our hierarchical model is found in Appendix S1.

Spontaneous variability in any marker over time combined with a fixed cutoff value for treatment decisions (such as initiating lipid-lowering treatment with statins based on CRP levels) implies that decision errors can occur. For example, using a cutoff value of 2 mg/L for CRP, someone with a true mean value below 2 mg/L and who the clinician may elect not to treat pharmacologically, may occasionally provide a value over 2 mg/L because of the random and generally unappreciated systematic variability inherent in any single measurement. We calculated the probability of such treatment errors (assuming that each individual does have a true mean value) by using an estimate of the individual between-month SD of CRP.

## Results

The 100 subjects were recruited over one year. Clinical characteristics of the 4 groups studied are presented in Table 1. Of the 1500 potential blood samples, there were only 8 missing samples, 3 from a subject who was imprisoned, 2 from a patient hospitalized with cellulitis, 1 due to weather conditions, and 2 from a subject who underwent hip surgery. Two subsequent CRP measurements in this latter patient were postponed by a few weeks each because of this event. These were the only postponements in CRP measurements due to a concomitant inflammatory condition. During the study, there was only one acute vascular event, an acute coronary syndrome in a subject of the recurrent events group that occurred midway between month-6 and month-9 blood draws.

**Table 1:** Clinical Characteristics of the 4 Study Groups.

GROUPS	1	2	3		4
	Recurrent Events (n = 25)	Single Remote MI (n = 25)	Longstanding AlwaysStable CAD (n = 25)		No CAD (n = 25)
Age (years)	65.6±8.4*	64.6±7.2	66.3±6.4		61.2±8.0
Sex (male)	88% (22)†	84% (21)	88% (22)		72% (18)
BMI (kg/m^2^)	29.9±4.1	28.6±3.0	28.4±3.4		29.4±5.2
BMI >30	52% (13)	28% (7)	28% (7)		38% (9)
Waist circumference (cm)	103.7±10.7	98.4±10.4	99.2±11.5		96.3±12.7
Current smoker	28% (7)	12% (3)	16% (4)		12% (3)
Ex-smoker	60% (15)	72% (18)	56% (14)		60% (15)
Never smoker	12% (3)	16% (4)	28% (7)		28% (7)
Type 2 diabetes	32% (8)	16% (4)	28% (7)		0%
Hypertension	80% (20)	48% (12)	72% (18)		52% (13)
Dyslipidemia	100% (25)	96% (24)	96% (24)		36% (9)
1^st^ cholesterol value (mmol/L)	3.81±0.94	3.94±0.47	3.96±0.72		4.79±0.94
History of renal failure	8% (2)	0%	4% (1)		4% (1)
Serum creatinine (µmol/L)	89.2±24.6	88.9±24.7	83.2±16.8		80.5±11.7
Urinary albumin/creatinine ratio	25.7±33.5	7.4±9.7	28.7±35.2		12.5±23.1
History of heart failure	32% (8)	0%	0%		0%
LV ejection fraction (%)	46±12	54±9	64±7		62±5
Duration CAD (years)	19.0±10.1	12.0±4.4	16.7±7.9		–
Stroke/TIA	4% (1)	0	4% (1)		0
Peripheral arterial disease	20% (5)	0	12% (3)		0
Ankle/brachial index	1.1±0.4	1.2±0.2	1.2±0.3		1.2±0.1
Medications					
Lipid-lowering drugs	96% (24)	96% (24)	92% (23)	40% (10)	
Angiotensin modulators	72% (18)	44% (11)	36% (9)	8% (2)	
Beta-blockers	88% (22)	68% (17)	72% (18)	28% (7)	
Aspirin/antiplatelet drugs	96% (24)	100% (25)	96% (24)	44% (11)	

* ± refers to standard deviation values;

† numbers of subjects in parentheses; MI = myocardial infarction; CAD = coronary artery disease; BMI = body mass index; LV = left ventricular; TIA = transient ischemic attack.

### Intergroup CRP Results

**Figure 1:**
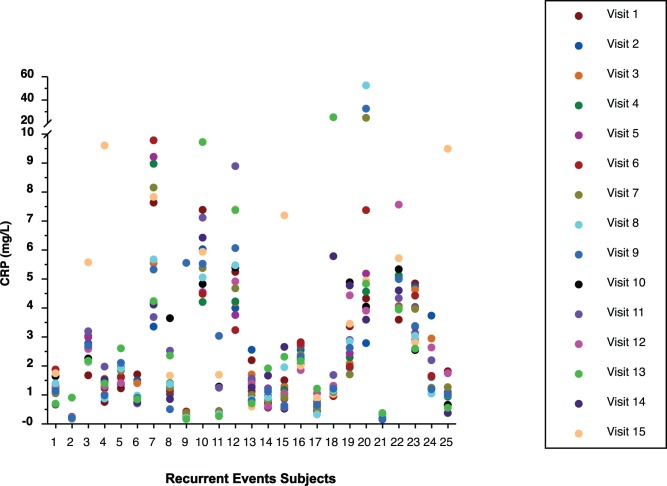
Display of all CRP values of subjects with recurrent acute coronary events.

**Figure 2:**
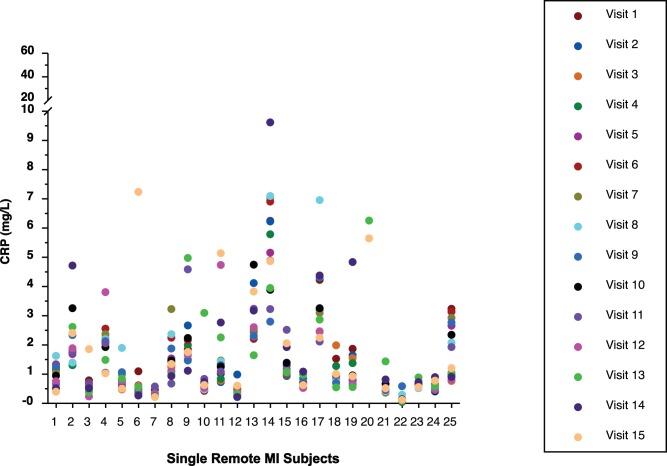
Display of all CRP values of subjects with a single remote myocardial infarction (MI).

**Figure 3:**
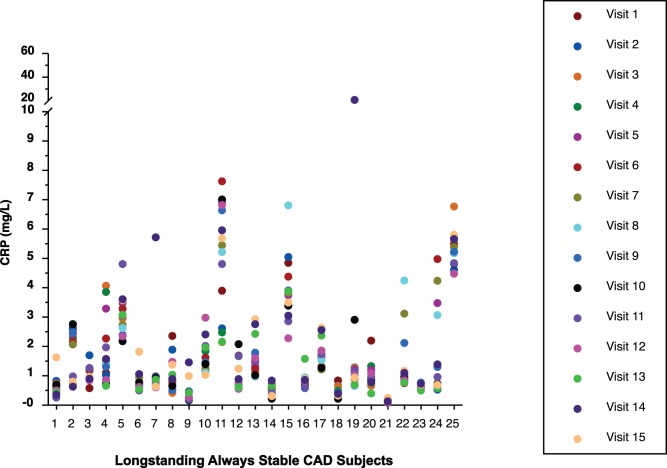
Display of all CRP values of subjects with longstanding always stable coronary artery disease (CAD).

**Figure 4:**
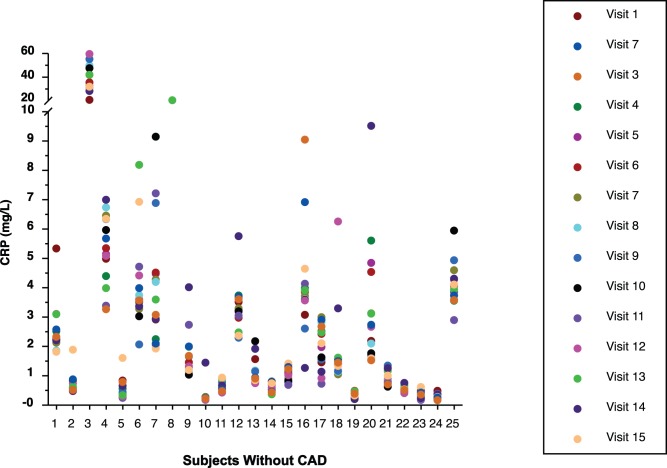
Display of all CRP values of subjects without coronary artery disease (CAD).

The 15 CRP values of all subjects by group are displayed in Figures 1, 2, 3, and 4. Median CRP values among the 4 groups were not clinically or statistically different (Table 2). Not only was there considerable overlap of CIs but the group without CAD had the highest median CRP while this group might normally have been expected to have the lowest CRP value, making it likely that these differences are not clinically meaningful. Because the pattern of CRP variability did not differ substantially among the 4 groups, all subjects were subsequently analyzed as a single group.

**Table 2:** Median CRP Values (mg/L ±95% CI) of the 4 Study Groups.

Recurrent Events (n = 25)	Single Remote MI (n = 25)	Longstanding Always Stable CAD (n = 25)	Group Without CAD(n = 25)
1.84 (1.14–3.00)	1.22 (0.78–1.78)	1.11 (0.81–1.92)	2.02 (1.03–2.95)

### Quantitative CRP Analysis

Using individual level SD estimates, the median SD values within-day, within-week, between-weeks and between-months CRP values were 0.07, 0.19, 0.36 and 0.63 mg/L, respectively. Estimating the SD parameter across subjects resulted in CRP SD values of 0.24, 2.03, 2.18 and 2.77 mg/L for within-day, within-week, between-weeks and between-months, respectively. The much larger values across subjects reflect widely differing mean values between subjects, which are eliminated in within-subject SD estimates.

Our hierarchical model estimated the global CRP mean to be 5.0 mg/L (95% CrI: 3.2, 7.0), with a between-subject SD of 1.8 mg/L (95% CrI: 1.4, 2.3). The presence of adjudicated inflammation status raised the mean by 0.3 mg/L (95% CrI: 0.1, 0.5). The effect of aspirin use and male sex lowered the CRP mean by 0.5 mg/L (95% CrI: −1.8, 0.6), and 2.6 mg/L (95% CrI: −4.1, −1.1), respectively, while increasing BMI raised the mean by 0.2 mg/L per BMI unit (95% CrI: 0.1, 0.4). Aspirin use, BMI, and sex also had small effects on the daily and weekly SD estimates. Other variables that were tried in the model to explain the variability of CRP included clinical group, left ventricular ejection fraction, and use of angiotensin modulators or lipid-lowering drugs, but these were eliminated from the final model, in large part because they were highly correlated with variables retained in the model, and so did not add sufficient additional predictive power.

### Qualitative CRP Overview Based on the 2 mg/L Risk Threshold

Of the 100 subjects, 35 had consistently low-risk CRP values (<2 mg/L) and 19 had consistently high-risk values (≥2 mg/L). The remaining 46 subjects changed risk category at least once during the study. Nineteen of them had a predominant low-risk pattern yet they had 1–4 exceptions in the high-risk range. Seven had a predominantly high-risk pattern yet they had 1–3 exceptions in the low-risk range. The remaining 21 of these 46 subjects had an inconsistent pattern with ≥4 values in both low-risk and high-risk ranges and this always included changes outside of the week with the 5 daily measurements.

**Figure 5:**
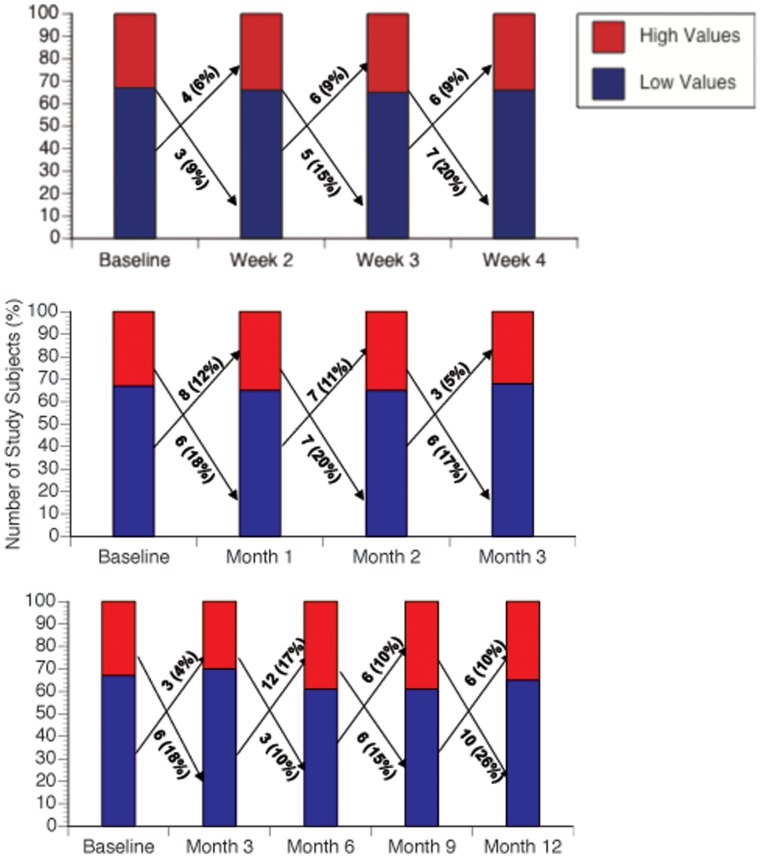
Numbers of subjects (and %) changing CRP risk category from one measurement to the next by weekly (top), monthly (middle), and tri-monthly (bottom) intervals.

The least variability was observed in the same day measurements. Based on the initial baseline morning measurement, only 2 subjects changed risk category at a subsequent measurement during the same day. The number of subjects who changed from high-risk to low-risk and from low-risk to high-risk categories on subsequent measurements within each serial time interval (over 5 days, 4 weeks, 3 months, tri-monthly) are shown in Table 3. A similar proportion of subjects in initial high-risk and initial low-risk categories changed to the other risk category on subsequent measurement in each series of time intervals. The number of subjects who changed risk category appeared to increase as the time interval of measurement increased, from 12 subjects (12%) in the daily measurements to 34 subjects (34%) in the tri-monthly measurements. In Figure 5, the changes from each measurement interval to the following one are illustrated graphically for the weekly measurements (times 4, including baseline), monthly measurements (times 4, including baseline), and 3-month intervals (times 5, including baseline).

**Table 3:** Numbers of Subjects Who Changed From Initial High Risk to Low Risk and From Initial Low Risk to High Risk Categories on Subsequent Measurements Within Each Serial Time Interval.

Interval	Consecutivesamplings	Subjects with initialhigh-risk CRP value	Subjects with change tolow risk on ≥1 subsequentmeasurement	Subjects with initial low-risk CRP value	Subjects with change to high risk on ≥1 subsequent measurement
6 Hour	3	33	1 (3%)	67	1 (1.4%)
Day	5	33	7 (21%)	67	5 (7%)
Week	4	33	7 (21%)	67	16 (24%)
Month	4	33	9 (27%)	67	14 (21%)
3 Month	5	33	12 (36%)	67	22 (33%)

### Results of Patient-Reported Symptoms & Events During the Study

There were a total of 1103 (73.9%) reported potential events that could impact on inflammation status at the 1492 blood draws of which 164 (14.9%) were adjudicated as clinically significant in their potential impact on concomitant CRP measurements. Most of these (84%) were judged of mild intensity. The remainders were judged of moderate intensity; none were considered to be of high intensity relative to the time of blood sampling.

We performed a sensitivity analysis to see whether and to what degree increases in CRP from one sampling visit to the next could be accounted for by patient-reported or adjudicated potentially inflammatory concomitant events. We found that symptoms and subject-reported events with potential impact on CRP measurements were poor discriminators of significant CRP rises. The adjudication process had some value but accounted for only a minority of the occasions when CRP increased substantially from one interval to the next.

### Probability of Making an Error of Risk Status Assignment

**Figure 6:**
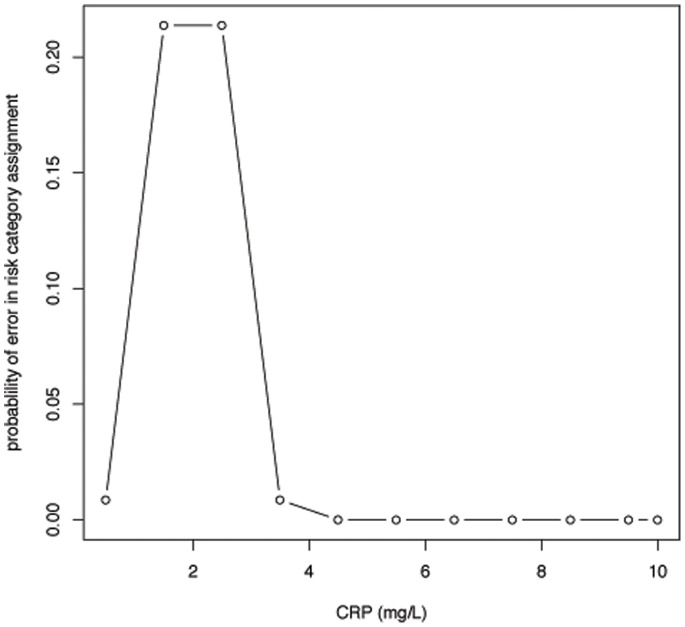
Probability of error in risk category assignment based on any assumed to be ‘true’ CRP measurement.

We calculated the probability of making an error in assigning a subject a low risk (<2 mg/L) versus high-risk (≥2 mg/L) category based on a single CRP measurement. We assumed a within-individual SD of 0.63 mg/L, which was the month-to-month variability. The probability of making an error is represented in Figure 6 as a function of a single CRP measurement. There is at least a 20% chance of an error in risk assignment if the true CRP value lies between 1.47 mg/L and 2.53 mg/L. The chance of an error is at least 10% for all true CRP measurements lying between 1.19 mg/L and 2.81 mg/L.

## Discussion

The principal findings of this study are: 1) CRP values and intergroup and intra-individual variability of CRP did not differ substantially among 3 distinct clinical subsets of patients with CAD and an age and sex-matched group without CAD; 2) On multiple and systematic daily, weekly, monthly, and tri-monthly measurements, CRP exhibited considerable intra-individual variability; 3) This random spontaneous variability persisted despite extensive efforts to control for systematic causes; 4) From the perspective of high-risk and low-risk assignment, 46% of the study subjects did not remain consistently within a single CRP risk category (based on a 2 mg/L cutpoint), even in the absence of any change in their cardiovascular status. Our focus is the individual patient in the clinical arena and what the clinician needs to know about the variability of absolute CRP measurements for clinical decision-making.

The individual between-months SD estimate of CRP was 0.63 mg/L, which is substantial for clinical decision-making with a risk threshold value of 2 mg/L. For example, an individual with a CRP measurement precisely at the high-risk cutoff of 2 mg/L may be expected on repeated sampling to have measurements that would lie between 0.74 mg/L and 3.26 mg/L (2 mg/L ±2 SD), considerably within both low-risk and high-risk ranges. As illustrated in Figure 6, an at least 20% chance of error in risk category assignment exists for individuals whose ‘true’ CRP value would lie between about 1.5 mg/L and 2.5 mg/L. A substantial proportion of the subjects in this study as well as the American population [26] have values within these limits. Moreover, in an individual participant meta-analysis of 160,309 subjects from 54 prospective studies, the median baseline CRP was 1.72 mg/L. [27] Similar findings have been shown in a multiethnic study of patients presenting with a first ST-elevation myocardial infarction. [28].

### Potential Drivers of CRP Variability

We found the least variability in the 3 diurnal CRP measurements but increasing variability over longer follow-up periods. One third of subjects, whether in the high-risk or low-risk CRP category on initial measurement, changed risk category on at least one subsequent tri-monthly measurement. The narrow variation of diurnal values is plausible since inflammation status would not usually be expected to vary over any one day in most individuals and attests both to the reliability of the measuring technique and the absence of a significant contribution of circadian variability. Most of the variability noted over the longer term could not be clearly accounted for by symptoms or events that were frequently reported by these stable subjects on systematic questioning regardless of any change in their CRP from one measurement to the next. Marked CRP variability was often presumably due to subclinical fluctuations in inflammation/infection status. It is likely that the variation noted in CRP values simply reflected these changes in inflammation status since in a previous study we found similar apparently spontaneous changes on serial measurement of the inflammation cytokine, interleukin-6. [29] Because of the plethora of variables besides infections, overt inflammation, and atherosclerosis that may affect the biomarkers of inflammation such as weight change, drugs, level of physical activity, changes in diet, smoking status, depression and trauma, and likely other indeterminate factors, it is not surprising that CRP values may exhibit swings that are often unpredictable.

### Previous Studies

Previous studies that have examined CRP variability have produced conflicting results. This inconsistency and the possible reasons to account for it have not been well addressed or debated. For example, in contrast to our findings, Ockene et al. [19] studied 113 healthy adults with 5 measurements of CRP and total cholesterol over one year and concluded that CRP had measurement stability similar to cholesterol. This conclusion is surprising because the authors reported a within-subject standard deviation of cholesterol of 0.447 mmol/L, which would be less than 10% a cut-off risk value of 5.2 mmol/L for cholesterol, while the within-subject standard deviation of CRP was 1.2 mg/L, which would be 60% of the cut-off risk value of 2 mg/L. Three other studies have suggested that CRP is relatively stable within individuals on serial measurement. The ‘stable’ values were measured over a period of 5–12 years and often excluded elevated CRP values without any clinical justification. [18], [21] More recently, Glynn et al. [23] studied CRP stability annually over five years in 8901 placebo-treated individuals within the JUPITER trial. Using box plots and correlation coefficients, the authors concluded that CRP in these individuals with high-risk initial values exhibits ‘strong tracking’ over the long term. However, because serial box plots track a group, the considerable fluctuation in serial measurements in the same individual could be obscured, if not cancelled out, when medians of a large group are examined. It may also be questioned whether the application of correlation coefficients on log-transformed data in this and the 2 preceding studies is the best means to analyze intra-individual stability. Log-transformation (that was applied to CRP but not to cholesterol) considerably attenuates the variance of the data. As well, correlations, especially non-parametric ones that mask outlying values, do not inform about the magnitude of the variability, but about how related measurements are, and hence are not a good means of understanding how CRP varies with time. These latter studies may thus considerably underestimate the variability of CRP over time.

In a recent individual participant meta-analysis that examined CRP and vascular risk, Kaptoge et al. [27] calculated regression dilution ratios and found in 22,124 subjects with 2 CRP measurements a mean of 5 years apart that CRP (log transformed) exhibited year-to-year intra-individual consistency similar to cholesterol (not log transformed) and systolic blood pressure. The design and methods of the study and its focus on outcomes do not address the problematic of variability encountered when CRP is used in daily clinical practice for risk stratification and individualized management based on a threshold risk value.

In contrast to these studies, others have suggested that CRP exhibits considerable variability with intra-individual coefficients of variation for CRP that are 4–5 fold larger than for cholesterol. [16], [17] Similar or greater variability of CRP has been found in other studies. [14], [15], [20], [22].

### Potential Limitations

Our study group was relatively small in comparison to some of the previous studies examining intra-individual CRP variability. However, previous studies have neither been as systematic in their design and analysis nor as intense in terms of numbers of measurements per subject and time-points as the current study. We used a total of 1500 observations to estimate variability of CRP over time. While that size is small compared to other studies that focused on outcomes, it clearly was an adequate size for estimating variance, as evidenced by our reasonably small interval estimates. The study group was highly selected; most had CAD and were on statin therapy. On the other hand, neither clinical group nor use of lipid-lowering therapy was retained in the hierarchical model of CRP variability. However, even if CAD status and statin use blunted CRP variability, this would suggest that CRP exhibits even more variability in the general population than was found in this study. Finally, our design and methods did not allow us to characterize intra-individual variability of CRP based on sex, age bracket, or ethnic group.

### Conclusion

Our study suggests that the use of CRP to assign an atherosclerotic disease risk status to individual subjects may be problematic. It cannot be assumed that a single value or even a pair of values will reliably define an individual’s stable or necessarily unchanging inflammation risk status. This does not detract from the importance of inflammation in the pathogenesis of atherosclerotic vascular disease or from its well-established epidemiological associations despite persistent controversy over its added value for risk stratification. In contrast to studies that have estimated the ability of CRP to predict future events averaged across tens of thousands of subjects, we have reported the individual level variation in day-to-day absolute CRP measurements, and subsequently the potential effect that this variability may have on predicting individual level future events. Our findings question the use of isolated CRP values to assign definitive risk status and to make long-term management decisions in individual patients in routine clinical practice.

## Supporting Information

Appendix S1 (PDF)
