# Supplementary material for: Time Variability of C-Reactive Protein: Implications for Clinical Risk Stratification
Source: PLoS One. 2013 Apr 8;8(4):e60759. doi: 10.1371/journal.pone.0060759 (PMC3620269; doi:10.1371/journal.pone.0060759)
Supplement: Appendix S1 — (PDF) [file pone.0060759.s001.pdf]

Statistical Appendix

Let X_{szdwm} represent the CRP value of subject s at observation time z on day d within week w during month m. Our five level hierarchical model can then be described as follows.

At the first level of the hierarchical model, we assume that CRP values within each individual follow a normal density within a given day, such that

$$X_{sz} \sim N(\mu_{sz}, \sigma^2_{sz}) \ ,$$

where μ_{sz} represents the overall mean of subject s depending on covariates z, and σ^2_{sz} represents subject-to-subject variability after accounting for variance explained by the covariates.

In turn, since covariates ASA, BMI and sex entered into our final model,

$$\mu_{sz} = \alpha_s + \beta_{ASA} \times ASA_s + \beta_{BMI} \times BMI_s + \beta_{sex} \times sex_s$$

where ASA_s, BMI_s and sex_s are the subject specific ASA, BMI and sex variables.

At the second level of our hierarchical model, we have

$$X_{szd} \sim N(\mu_{szd}, \sigma^2_{szd}) \ ,$$

where μ_{szd} represents the mean of subject s on day d, and σ^2_{szd} represents the variance within subjects on day d.

At the third level of our hierarchical model, we have

$$X_{szdw} \sim N(\mu_{szdw}, \sigma^2_{szdw}) \ ,$$

where μ_{szdw} represents the mean of subject s within week w, and σ^2_{szdw} represents the week-to-week variance within subjects.

At the fourth level of our model, we have

$$X_{szdwm} \sim N(\mu_{szdwm}, \sigma^2_{szdwm}) \ ,$$

where μ_{szdwm} represents the mean of subject s within month m, and σ^2_{szdwm} represents the month-to-month within subject variance.

Finally, our model is rounded out by assuming low-information prior densities across all parameters. In particular, α_s and all regression parameters β

followed normal densities with zero means and large variances, and standard deviation parameters were uniformly distributed across a range that included all plausible values. We also ran a simpler model without regression covariates ASA, BMI and sex, and a somewhat more complex model that added a regression component to explain subject-to-subject differences in variance. This latter model also included ASA, BMI and sex as covariates.
